# Creating a Three-Dimensional Reconstruction of the Glenohumeral Joint From Magnetic Resonance Imaging to Assist in Surgical Decision-Making

**DOI:** 10.1016/j.eats.2024.102972

**Published:** 2024-04-09

**Authors:** Jacob N. Dowe, Matthew W. Bradley, Lance E. LeClere, Jonathan F. Dickens

**Affiliations:** a Department of Orthopaedic Surgery, Walter Reed National Military Medical Center, Bethesda, Maryland, U.S.A.; b Uniformed Services University of the Health Sciences, Bethesda, Maryland, U.S.A.; c The Geneva Foundation, Tacoma, Washington, U.S.A.; d Department of Orthopaedic Surgery, Naval Health Clinic Annapolis, United States Naval Academy, Annapolis, Maryland, U.S.A.

## Abstract

Understanding the anatomical structure of a patient’s shoulder joint is essential in surgical decision-making, especially regarding glenohumeral bone loss. The use of various imaging techniques, such as magnetic resonance imaging (MRI) and computed tomography (CT), bring certain advantages and disadvantages in assessing joint structure. Before a surgical procedure, bone loss can be observed and measured using these imaging techniques in both 2-dimensional and 3-dimensional (3D) views. The ability to visualize the shoulder joint in a 3D manner, as commonly done with CT scans, is helpful in assessing bone loss; however, CT involves exposure to radiation, additional time, and greater costs. The process of obtaining a 3D view of the shoulder joint from an MRI, although less common, can be completed effectively to assess bone loss while also solving some issues surrounding CT scans. By loading MRI datasets into an image-reformation program, such as 3D Slicer, the anatomical structures can be segmented to create realistic 3D models of the shoulder joint. Surgical direction can be determined after bone loss measurements and structural assessment of these models, without the need for CT scans. This technique can also be applied to other skeletal joints, in addition to the shoulder.

For patients presenting with shoulder injuries potentially requiring surgery, particularly following instability events, current standard of care includes a magnetic resonance imaging (MRI) scan for the assessment of soft-tissue pathology and bone loss.^1^, ^2^, ^3^, ^4^, ^5^, ^6^, ^7^ MRIs produce 2-dimensional images of varying axes to understand the presence and severity of the patient’s injuries. Although MRIs allow for excellent soft-tissue assessments, the assessments for bone loss are not as strong as compared with other imaging modalities, such as computed tomography (CT).^1^^,^^6^^,^^8^, ^9^, ^10^ Given that the degree of bone loss can alter the surgical direction chosen, obtaining accurate and detailed images of the bony structures is vital in the patient care process.^7^^,^^10^^,^^11^

The closest to a current gold standard for assessing bone loss in the shoulder is by obtaining a 3-dimensional (3D) CT scan and then using a 3D reconstruction of the structures.^1^^,^^2^^,^^6^, ^7^, ^8^, ^9^, ^10^, ^11^, ^12^, ^13^ The ability to visualize the shoulder joint in a 3D manner, as commonly done with CT scanning, is helpful in assessing the degree of bone loss; however, CT scans expose patients to radiation, are limited by cost and availability, and require additional time commitments from patients, clinicians, and radiology technicians.^1^, ^2^, ^3^, ^4^^,^^6^, ^7^, ^8^^,^^11^^,^^13^, ^14^, ^15^, ^16^, ^17^ In addition, CT does not allow for the assessment of intra-articular soft tissue and chondrolabral pathology.^2^^,^^6^

The process of obtaining a 3D view of the shoulder joint from an MRI, although less common, can be completed by including an additional brief (∼3 minute) scan sequence to the MRI protocol, such as the 3D spoiled gradient recalled echo sequence. Parameters used on a GE 750W 3T scanner (GE Healthcare, Chicago, IL) are outlined in Table 1. Replacing the use of CT scans with an efficient and effective alternative that is already part of the patient’s care would benefit patients by eliminating the downsides of CT scans while also assisting clinicians in the determination of surgical direction through proper imaging detailing any injuries.^1^^,^^3^^,^^4^^,^^7^, ^8^, ^9^, ^10^, ^11^^,^^13^^,^^15^, ^16^, ^17^, ^18^

**Table 1:** 3D SPGR FS MRI Scan Parameters

Parameter	3D SPGR FS
Plane	Oblique COR
Imaging mode	3D
Pulse sequence	SPGR
Imaging options	Fast, ARC, Zip512
Field of view, cm	16
Slice thickness, mm	1.2
Spacing	0
Number of slices	76
Number of echos	1
Flip angle, °	16
Repetition time	Minimum
Echo time	Minimum
Receiver bandwidth, kHz	50
Frequency, MHz	288
Phase	192
Frequency direction	S/I
NEX/ACQ	1
Fat/water saturation	FAT
Auto shim	Auto
Phase correction	Off
Coil filter	None
Time	3:33 minutes

3D, 3-dimensional; COR, coronal; NEX/ACQ, number of excitations/acquisitions; SPGR FS, spoiled gradient recalled echo sequence fat-saturation.

This Technical Note describes a process for creating 3D reconstructions of the shoulder joint. The approach outlined in this article allows for the development of an effective and efficient technique for learning valuable clinical information, especially bone loss assessment. By developing a 3D reconstruction from MRI scans as opposed to CT scans, it is believed that improvements can be made in patient care and surgical decision making. This study was approved by the institutional review board at Walter Reed National Military Medical Center.

## Surgical Technique (With Video Illustration)

This technique uses a program called 3D Slicer, which is an open-source, free, computer-based image viewer and data reconstruction platform (Version 5.2.2)^19^^,^^20^ (Video 1). Common terminology and related definitions may be found in Table 2. Information regarding navigation of the software is present in Table 3.

**Table 2:** Definitions of Common Terminology

Segments	Term used to describe the anatomical structures or sections after they have been separated from other structures, characterized by specific identifiers such as name and color.
Seeds	The paint markings on the image scans that are used to identify and categorize segments.
Reconstruction	The process of transforming an image scan, for example, a 2D-MRI or CT scan, into a 3-dimensional structure, based on the original data.
DICOM files	Patient imaging data used to assess structures and develop reconstructions.
Structures	The area of focus of the scan before segmentation. For example, bones, muscles, and soft tissue.
Master volume	The loaded scan data set that is used to produce reformations of the scan in various planes.
Reformatting	The process of changing a normal or standard image into a different angle, view, orientation, size, or display. The process of changing the characteristics of the original data set into a different form of representation.

2D, 2-dimensional; CT, computed tomography; DICOM, Digital Imaging and Communications in Medicine; MRI, magnetic resonance imaging.

**Table 3:** Image Navigational Instructions

MRI view	
Scroll	Changes MRI slice
Shift + scroll	Changes diameter of brush
Command + scroll Right click + move cursor	Zoom in/out of image
Shift + left click	Drag/move MRI scan image around screen
Left click + move cursor	Paint/draw/erase
3D view	
Click + move cursor	Free movement
Shift + left click	Drag/move model around screen
Command + click	Rotate model
Scroll Control + move cursor	Zoom in/out of model
Any view	
Shift + move cursor	Adjusts the view of the other planes to match the location of the cursor

MRI, magnetic resonance imaging.

### Download and Open 3D Slicer

**Fig 1:**
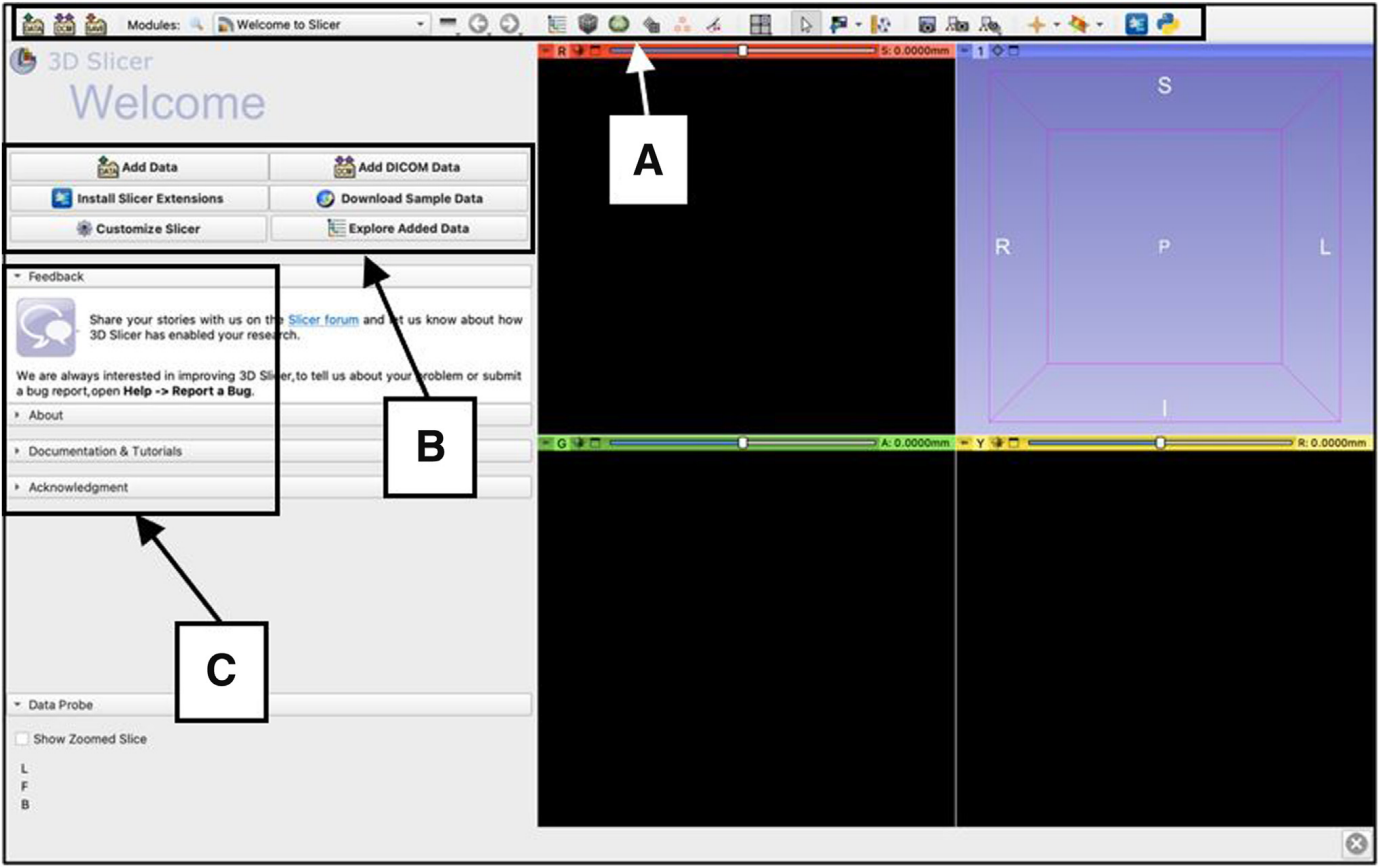
Standard display of the home page of the 3D Slicer application on a computer with macOS software. This is the starting point that is seen before using the application for the manual segmentation of magnetic resonance imaging to create a 3-dimensional reconstruction. The toolbar on the screen can also be used to load and save data, change modules, change views, screenshot image views, along with a variety of other functions (A). New and sample data can be loaded, and extensions can be installed from this page (B). Documentation and tutorials on how to use the program, along with other informational sections, are also available (C).

The 3D Slicer program can be downloaded onto any computer with Windows, macOS, or Linux software. After the software is downloaded and installed, navigate to the 3D Slicer icon and open the application. The home page will be a ‘Welcome to Slicer’ page (Fig 1).

### Import Digital Imaging and Communications in Medicine Data and Load Images

**Fig 2:**
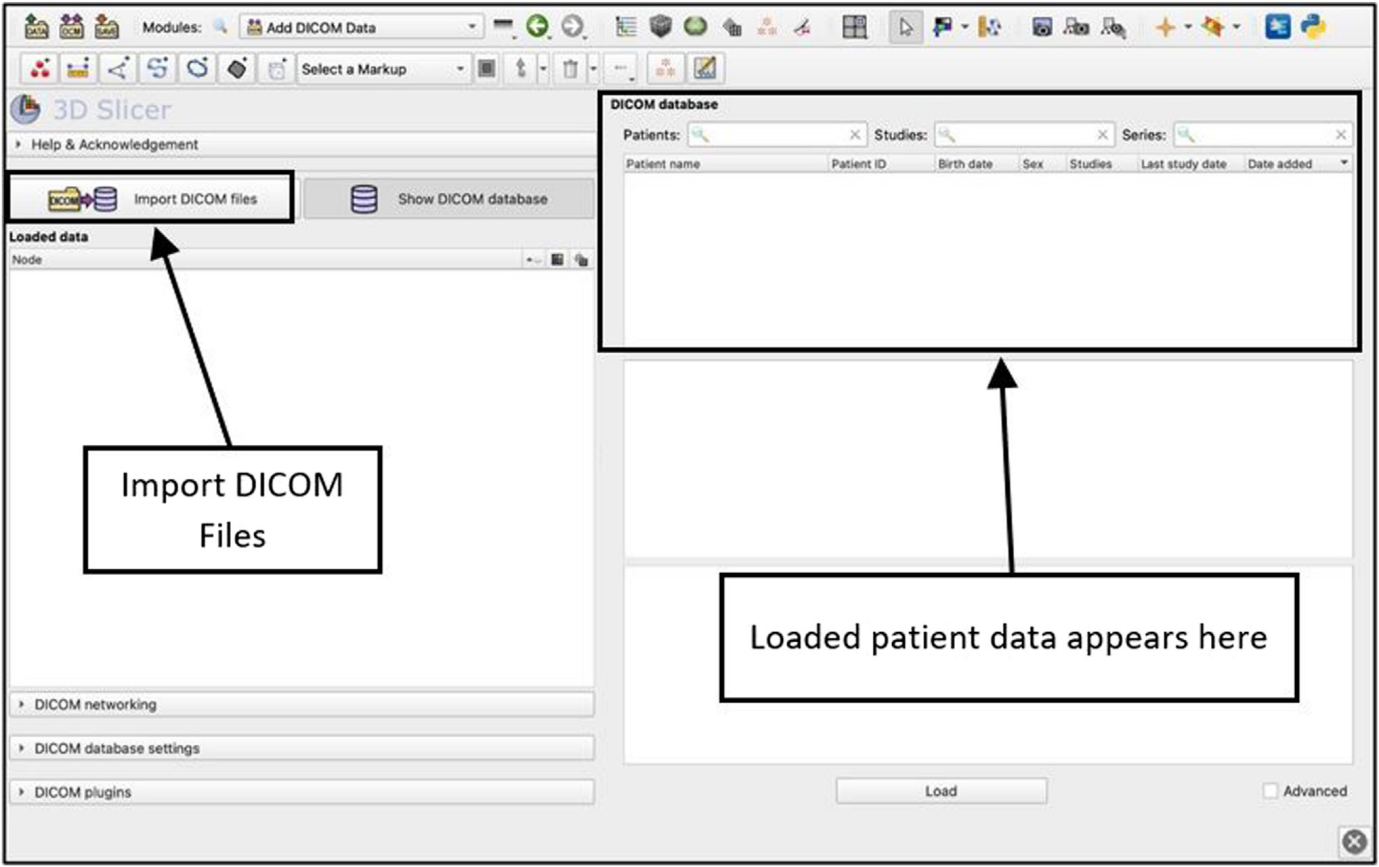
View of the DICOM module of the 3D Slicer application. The ‘DICOM’ module is where files can be imported, and the database of scan sets is stored. This page may act as the storage repository for the imported magnetic resonance imaging files. Selecting files from this page will load and prepare them to be edited for 3-dimensional model reconstruction. (DICOM, Digital Imaging and Communications in Medicine.)

View the Digital Imaging and Communications in Medicine database (Fig 2) and select the specific image dataset from which a reconstruction will be developed. This will be the master volume. Load the data and the software will use the master volume to create reformatted images. Image views should appear in the axial, coronal, and sagittal planes. A window for the 3D reconstruction will also be present. The purpose of this window is to view and rotate the image planes and view the reconstruction. Different master volumes may produce reformations that vary in quality, so it is important that the selected master volume shows clear images of the structure being studied. Various options exist that may improve image and segmentation quality.

### Open Segment Editor and Rename Segments

**Fig 3:**
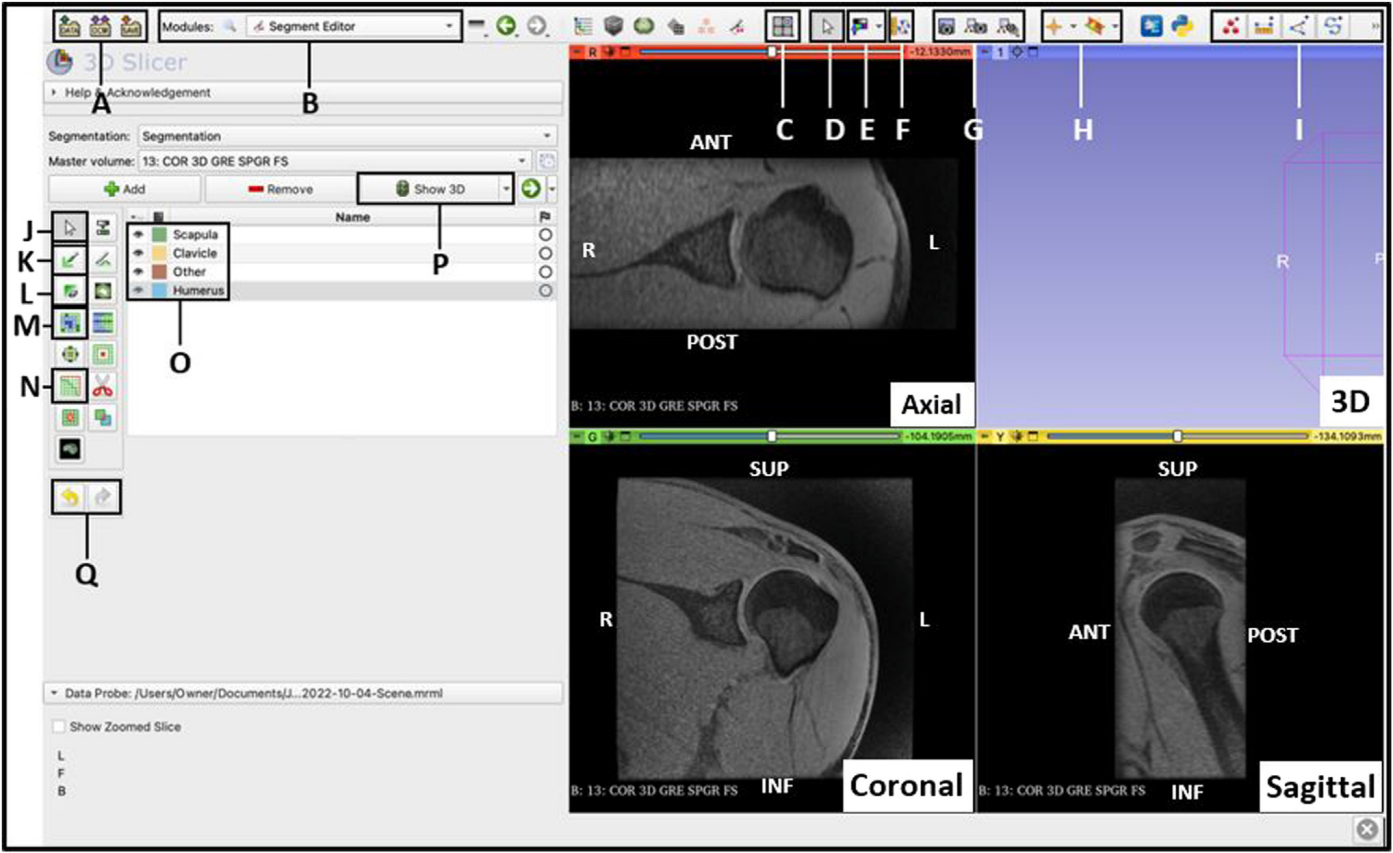
View of the Segment Editor module of the 3D Slicer application, with a patient’s left shoulder 3D spoiled gradient recalled echo functional sequence magnetic resonance imaging scan automatically displayed in the axial, coronal, and sagittal planes. The 3D view is empty as no model has been created yet. Each segment that will be displayed is listed, named, and assigned a color (O). This module provides the tools, functions, and effects that may be used or applied when editing and modifying the segments. Listed are the locations of popular functions used when creating a 3D reconstruction: (A) Load and Save Data; (B) Module List; (C) Change View; (D) Adjust View; (E) Adjust Image Volume; (F) View Markups Toolbar; (G) Record Screenshot of View; (H) View Image Crosshairs; (I) Markups Toolbar (Points, Lines, Angles, Curves, Measurements); (J) No Editing Selection; (K) Paint Effect; (L) Erase Effect; (M) Grow from Seeds Effect; (N) Smoothing Effect; (O) Segment List with Colors and Visibility Option; (P) Show 3D Reconstruction; (Q) Undo and Redo (3D, 3-dimensional; ANT, anterior; INF, inferior; L, left; POST, posterior; R, right; SUP, superior.)

Navigate to the ‘Segment Editor’ module and add a segment for each structure in the scan that will be separated (Fig 3). Name the segments accordingly and select a color by which to identify the structures. The segments can be as specific as desired regarding different features of the joint; however, for the purpose of completing a general segmentation of the glenohumeral joint, the main segments should include the scapula, the humerus, and the clavicle. In addition, add a final segment, and label it as ‘other’ to allow for proper segmentation by separating any structures that are not the focus of the segmentation.

### Paint Structures by Segment

**Fig 4:**
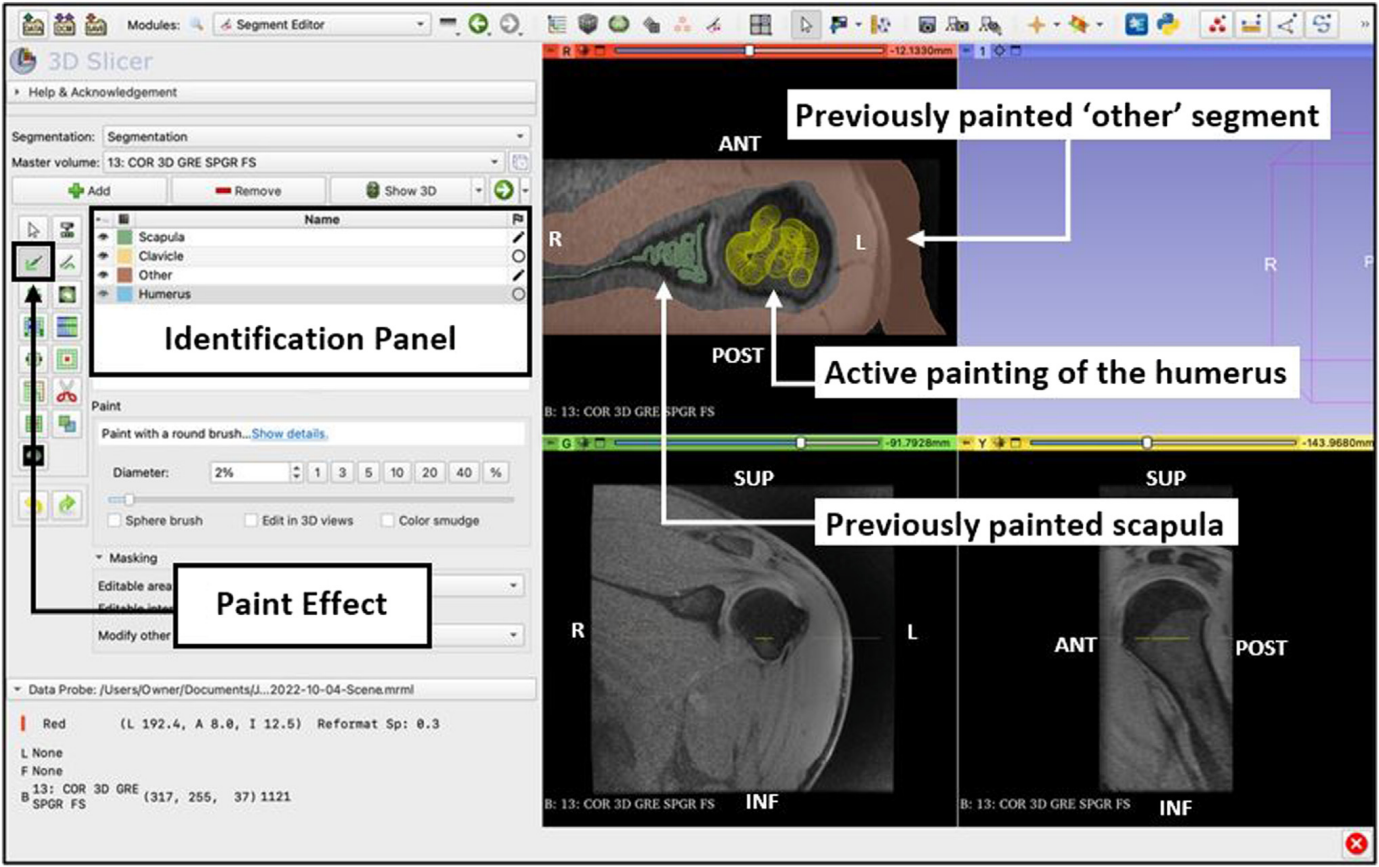
Use of the ‘paint’ effect from the Segment Editor module view of the 3D Slicer application. The cursor is used to mark the structures with their respective color as indicated on the Identification Panel. The actively selected segment (humerus) will display the cursor as a yellow circle and track the area being painted. Once the scan has been painted, the appropriate segment color is displayed (Scapula and Other). Accuracy of the reconstruction is improved as more slices and viewing planes are painted. (ANT, anterior; INF, inferior; L, left; POST, posterior; R, right; SUP, superior.)

Select the ‘paint’ effect and draw over each segment’s structure with its respective color (Fig 4). For example, select the ‘scapula’ segment, and draw over the scanned images of the scapula. For developing a model with the focus of assessing bone loss, structures such as muscle, tendons, air, and other soft tissue can be painted as part of the ‘other’ segment. Mark each structure with its respective color at various slices of the scan in each of the different viewing planes, as this will help the software recognize and connect similar structures. The segments do not need to be colored completely, just enough so that the structure is recognizable and identified by the program. Each paint marking that is applied is known as a ‘seed.’

### Grow From Seeds

**Fig 5:**
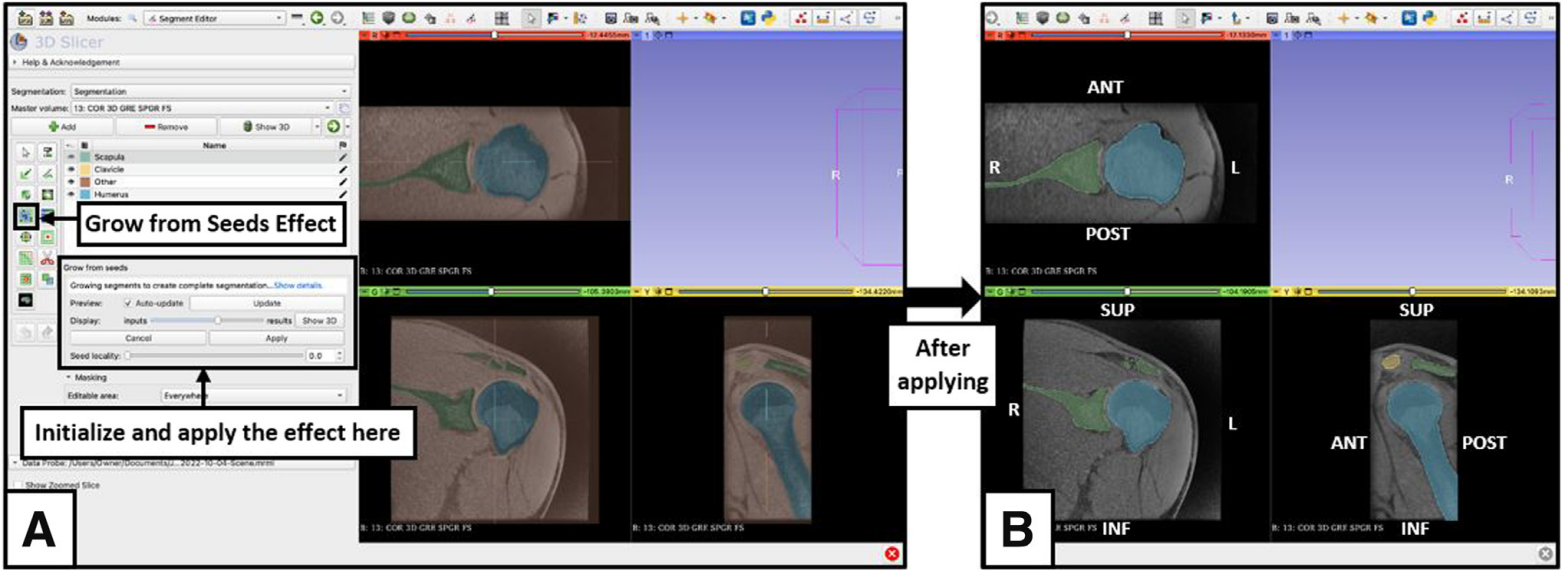
Use of the ‘grow from seeds’ effect from the Segment Editor module view of the 3D Slicer application. (A) Preview; (B) Applied. The ‘Grow from Seeds’ effect allows the computer software to analyze the painted sections of the scan, extrapolate, and apply the colors of different segments to areas of similar intensity, based on location, size, and shape. In a scan of good quality, this will separate the structures from each other and show the segments colored in the entire scan. Ideally, the segments should be outlined by the appropriate color; however, quality, contrast, and magnetic resonance imaging data set sequencing may impact the validity of the segmentation. (ANT, anterior; INF, inferior; L, left; POST, posterior; R, right; SUP, superior.)

Select the ‘grow from seeds’ effect on the Segment Editor module (Fig 5). Indicate visibility of the seeds of each segment by selecting the eye icon, and initialize the process. With the segments separated by color, slide the viewing scale at different slices and check the accuracy of the effect. Paint over areas that are identified by an incorrect color with the correct color. The ‘Auto-update’ function will show the proper segmentation with painted seeds. Selecting ‘Show 3D’ will preview the model to assist with determining accuracy. If the data set looks like it is segmented well, apply the effect, and the scan will be segmented.

### View 3D Model

Select the ‘Show 3D’ feature in the Segment Editor module to show a 3D reconstruction of the scan segmentation in the 3D viewing panel. Remove the ‘other’ segment by selecting the eye icon, and the structures of focus should be revealed.

### Smoothing

**Fig 6:**
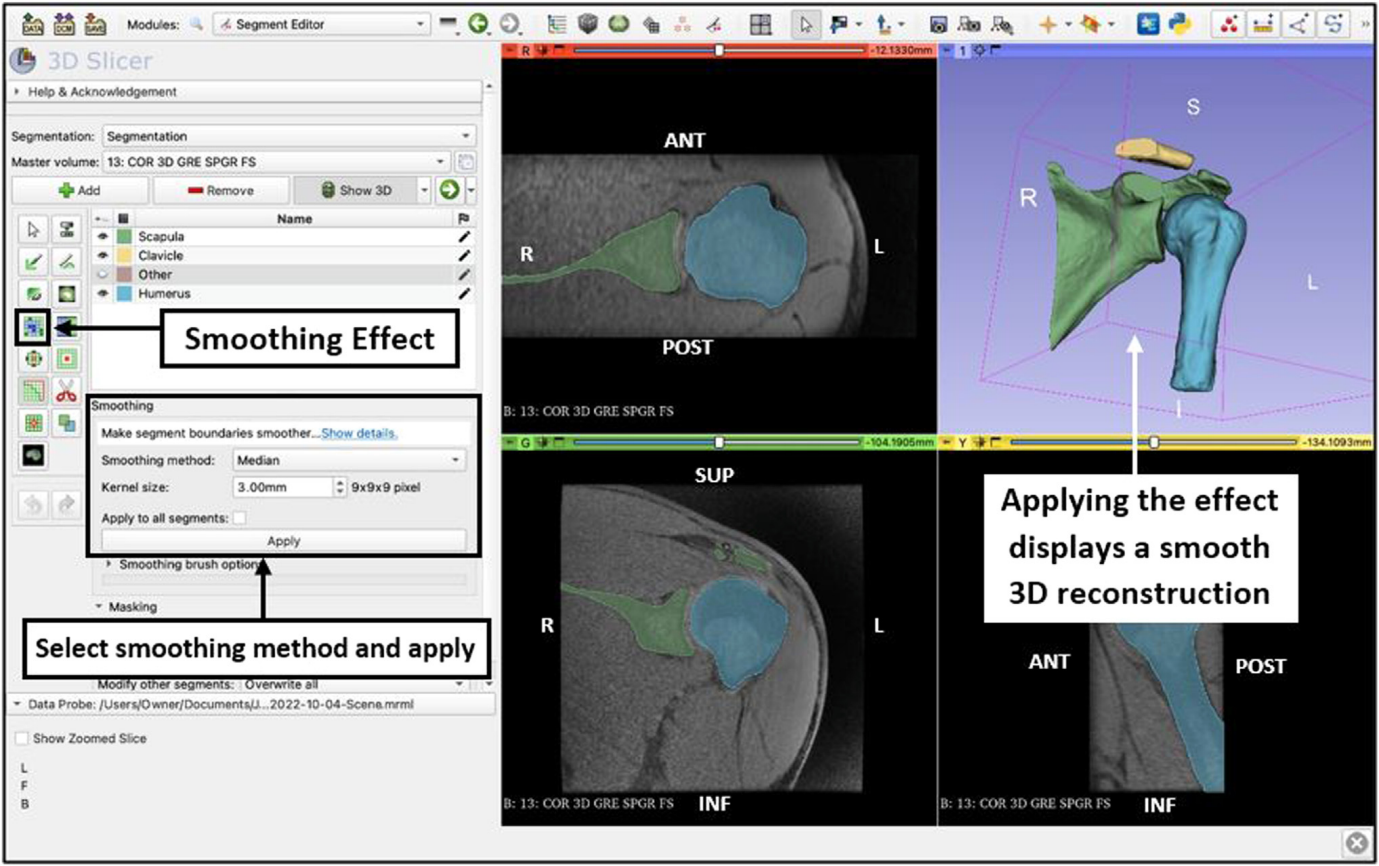
Use of the ‘smoothing’ effect from the Segment Editor module view of the 3D Slicer application. The ‘Median’ smoothing method is selected and applied to reduce minor extrusions as a result of artifact noise from the scan. A smooth version of the reconstruction is automatically displayed in the 3D View panel after applying the effect. (ANT, anterior; INF, inferior; L, left; POST, posterior; R, right; SUP, superior.)

Select the ‘smoothing’ effect (Fig 6) and choose the smoothing method that bests suits the goal of the reconstruction. Adjust the smoothing factor to change the intensity of the effect. This should only be used sparingly to reduce noise artifact from scan quality, when recording measurements, since validity and accuracy may be reduced as a result of misrepresentation of the anatomical structures.

### Fill Holes/Remove Extrusions

**Fig 7:**
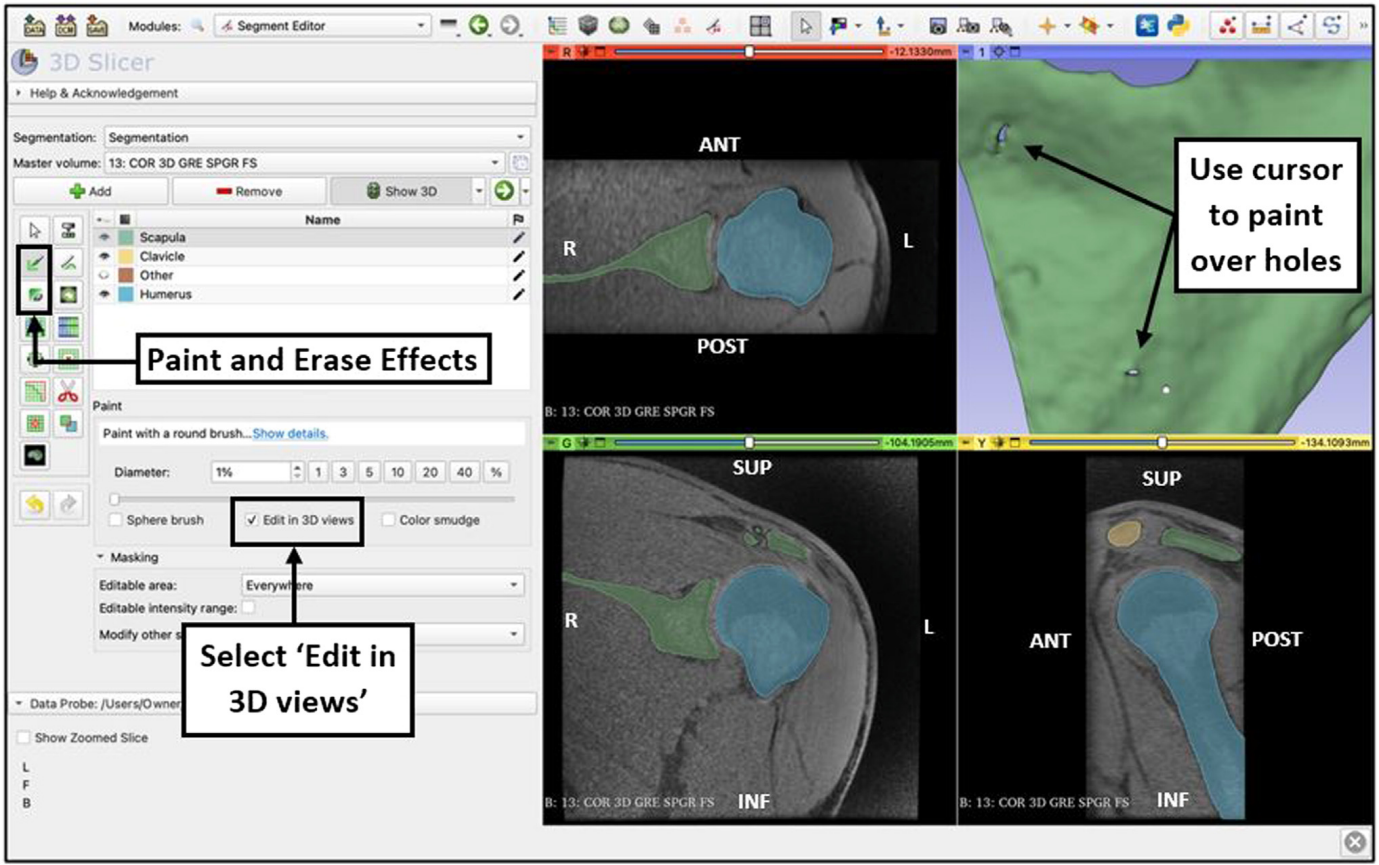
Use of the ‘paint” effect from the Segment Editor view of the 3D Slicer application to fill holes or remove extrusions on the 3D reconstruction view. The ‘Paint’ effect is used to fill small holes that may be present from areas of segments not being correctly identified with the proper segmentation color. The ‘Erase’ effect is used to clean and remove any extrusions (not shown) that may be present from areas being marked by an incorrect segmentation color. The cursor may be used in either the 2-dimensional or 3-dimensional views to achieve the desired outcome, creating a proper, realistic representation of the bone segments. (ANT, anterior; INF, inferior; L, left; POST, posterior; R, right; SUP, superior.)

If any holes appear in the reconstruction, select the paint effect and either add painted seeds on the scanned images, or paint directly on the model with ‘Edit in 3D views’ selected (Fig 7). Use the ‘erase’ effect to remove sections. If holes and extrusions are present at sections of the structures that do not change measurements, this effect may not be required.

### Reveal Completed Model and Record Measurements

**Fig 8:**
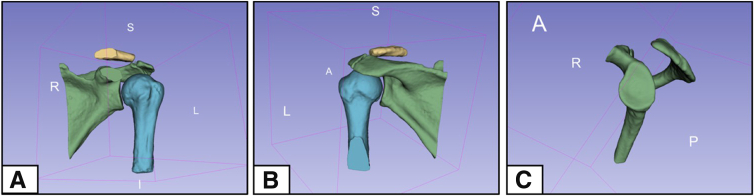
Views of a 3D model reconstruction developed from a 3D magnetic resonance imaging scan, completed using the 3D Slicer application. Present are the scapula (green), humerus (blue), and clavicle (yellow) on a left shoulder, in the anterior (A), posterior (B), and en face glenoid (C) perspectives. (3D, 3-dimensional; A, anterior; I, inferior; L, left; P, posterior; R, right; S, superior.)

In the 3D viewing panel, a final 3D reconstruction should be present. Fix or correct any errors as necessary. Adjust the model by rotating and moving it, and remove segmentations to view the structures at various angles (Fig 8). Save the file, or specific images of interest, using the toolbar. Apply measurement techniques to compare various models in order to determine glenohumeral bone loss, and analyze the data.

## Discussion

Bone loss measurements are essential in surgical decision-making. Viewing a proper, realistic model allows for a better conceptualization of structural differences. With the use of 3D reconstructions made from an MRI scan, as opposed to a CT scan, patient care can be improved and the process of determining surgical direction can be enhanced. The technique presented in this paper enables individuals to create 3D reconstructions and manipulate images for measurements, from the use of MRIs. The main focal areas of this process include obtaining the proper MRI scan that is both suitable for the software and the patient, as outlined, understanding and implementing the main functions of the effects of the reconstruction software, and developing a realistic, valid model that measurements can be collected from. Using the proper protocols and resources, this technique can portray anatomical information, contribute to clinical understanding, and improve surgical decision making relative to the glenohumeral joint.

It is essential to continue to enhance the aspects that contribute to surgical planning and decision-making, as well as the development of educational tools. Use of 3D imaging and modeling as a concept in the field of orthopaedic surgery is prevalent and continuing to grow in achieving this goal.^10^^,^^18^^,^^21^, ^22^, ^23^, ^24^ The technique described in this article may also be beneficial for other skeletal joints and structures as well. Similar techniques and use of 3D scans for analysis and model reconstruction have been shown to be accurate, educational, and beneficially impactful in improving outcomes when used for joints such as the knee.^21^, ^22^, ^23^, ^24^

Currently, MRI scans are primarily valued for their standard use and success in evaluating soft-tissue injuries and determining associated treatment plans with less of a focus on bone loss, as this effort usually goes to that of the CT scan.^1^, ^2^, ^3^, ^4^, ^5^, ^6^, ^7^^,^^9^, ^10^, ^11^^,^^15^, ^16^, ^17^ CT scans, usually done in addition to MRIs, can provide structural and quantitative information, but at the cost of radiation, and limitations of time, financial expenses, and availability.^1^, ^2^, ^3^, ^4^^,^^6^, ^7^, ^8^^,^^11^^,^^13^, ^14^, ^15^, ^16^, ^17^ Clinician assessment and surgical direction are often informed by 3D reconstructions from CT scans, but a 3D MRI scan essentially has the ability to replicate this.^1^^,^^3^^,^^6^, ^7^, ^8^, ^9^^,^^15^^,^^16^

The main factor that impacts the success of this process is the quality and type of the MRI scan. A good quality scan can decrease other limitations, such as the time spent completing the reconstruction process, and the accuracy of the model. A 1.5-T scanner may suffice regarding quality, depending on the purpose of the reconstruction; however, a 3-T scanner may develop the best images. Using an appropriate sequence, such as the 3D spoiled gradient recalled echo sequence, is valuable as well, and that is limited as this sequence cannot be completed after an MRI arthrogram is performed. The 3D aspect of the scan, as opposed to traditional 2-dimensional MRI scans, and the sequence type are both major contributors to the success of the reconstructions. 3D scans with the ability to visually separate bone from soft tissue, create an accurate and representative model, and develop a realistic understanding of anatomical interactions greatly enhanced this success.^7^^,^^18^^,^^22^, ^23^, ^24^ The length of the process varies with experience and purpose, as familiarity of the software and level of detail may adjust efficiency. Availability of other software to analyze and record measurements may be a limitation as well. Understanding the limitations and developing an efficient plan based off of the resources available allows proper value of the technique and reconstruction process.

The overarching goal is to identify a plan of care that can help providers with their ability to deliver patients with the most effective and efficient care possible. With MRIs already often used, no additional risks are involved, and by replacing CT scans for the purposes described, limitations may be removed. With future research and application, a collective approach can lead to development, standardization, and technique optimization. A new standard of care can emerge that improves the assessment of bone, mediates the risks and limitations with current practices, and has the potential to accomplish the goal of enhanced patient care.

## Disclosures

The authors report the following potential conflicts of interest or sources of funding: This work was supported in part by the 10.13039/100007188Uniformed Services University, Department of Physical Medicine & Rehabilitation, and Musculoskeletal Injury Rehabilitation Research for Operational Readiness (MIRROR) (HU00011920011). All authors (J.N.D., M.W.B., L.E.L., J.F.D.) declare that they have no known competing financial interests or personal relationships that could have appeared to influence the work reported in this paper. Full ICMJE author disclosure forms are available for this article online, as supplementary material.

## Supplementary Data

ICMJE author disclosure forms

ICMJE author disclosure forms

ICMJE author disclosure forms

ICMJE author disclosure forms

Video 1 This video outlines and describes the process of converting a coronal, 3-dimensional, spoiled gradient recalled echo functional sequence MRI scan of the left shoulder into a 3-dimensional virtual model reconstruction, using the computer software 3D Slicer. With loaded patient data, use of the Segment Editor module is explained. The process shown includes adding segments (scapula, clavicle, humerus, and other), using the ‘paint’ effect, using the ‘grow from seeds’ effect, reviewing the accuracy of the segmentation, using the ‘smoothing’ effect, and viewing, editing, and analyzing the model in a 3-dimensional view. The segments added are listed separately and provided with a name and color to represent the various structures in the scan (green = scapula; yellow = clavicle; red = other; and blue = humerus). The ‘paint’ effect is used to mark the segments in the different views of the loaded MRI scan data with the respective color to allow the computer to recognize specific features. The ‘grow from seeds’ effect uses the painted seeds to determine structures based on computer processing. Reviewing the segmentation after use of these effects allows for editing changes to be made and allows an accurate and valid representation of the MRI dataset. The ‘smoothing’ effect enhances the view of the model and shows a realistic form, removing minor image noise from the scan. Visualizing and editing the model in the 3-dimensional view fixes errors in the segmentation and allows for proper reconstruction. After a final 3-dimensional model reconstruction is completed, the model file or images can be saved, and the information can be used or applied to measurement records or bone loss analysis. The resulting information from an analysis can be used as part of the process of determining surgical direction when understanding structural characteristics, measurement data, and biomechanical ability. (MRI, magnetic resonance imaging).
